# Supplementary material for: YBX1 integration of oncogenic PI3K/mTOR signalling regulates the fitness of malignant epithelial cells
Source: Nat Commun. 2023 Mar 22;14:1591. doi: 10.1038/s41467-023-37161-0 (PMC10033729; doi:10.1038/s41467-023-37161-0)
Supplement: Supplementary file 1 — Supplementary Information [file 41467_2023_37161_MOESM1_ESM.pdf]

SUPPLEMENTARY FIGURES

Figure S1: Gene set enrichment for EMT and PI3K/AKT/mTOR signalling in the TCGA-HNC cohort

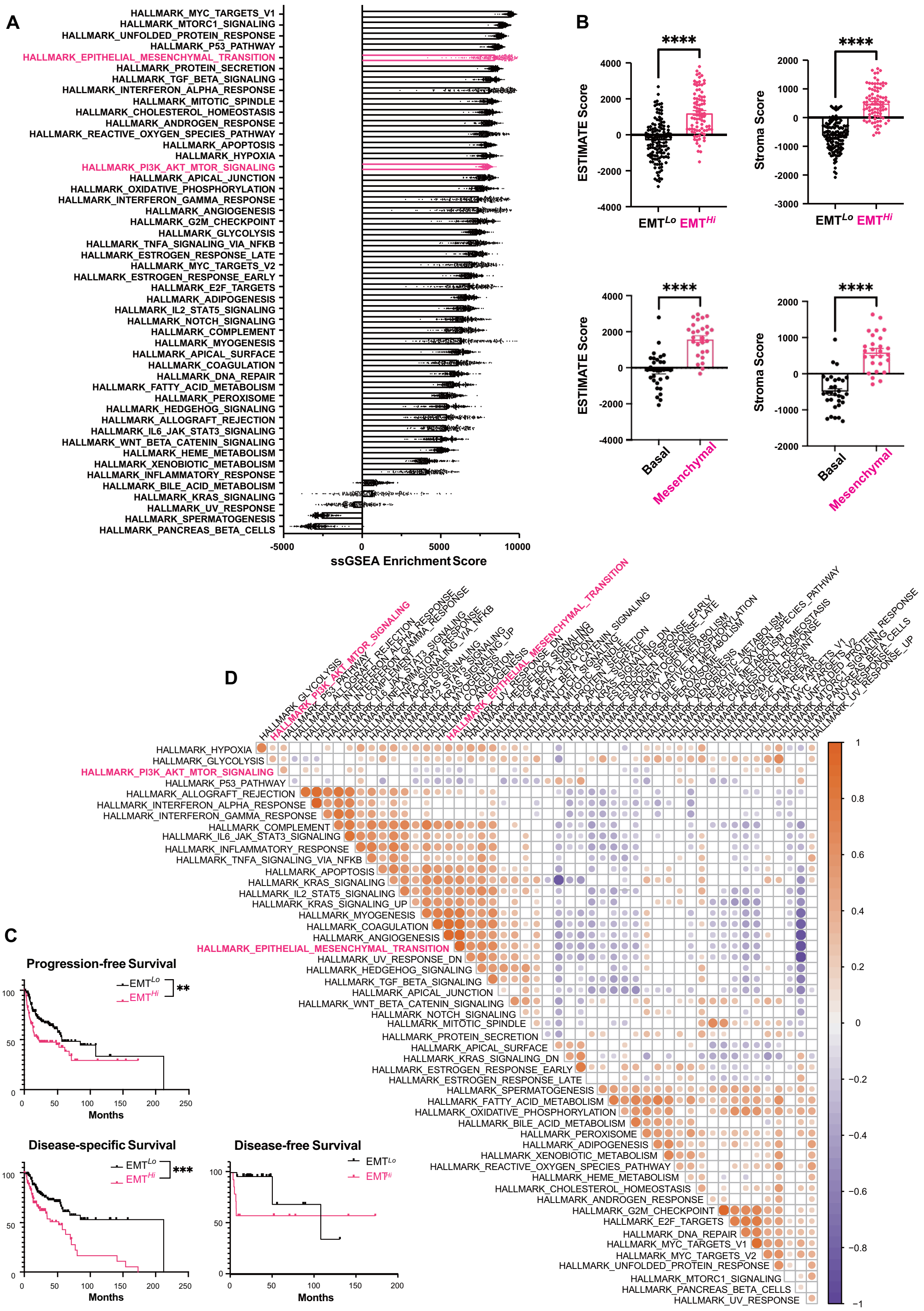

Figure S1. Gene set enrichment for EMT and PI3K/AKT/mTOR signalling in the

TCGA-HNC cohort. A. Overview of ssGSEA scores of hallmark genes in TCGA-HNC samples (n=211). **B.** ESTIMATE and Stroma scores were generated using expression datasets from the TCGA-HNC cohort (Basal n=31, Mesenchymal n=27, EMT^{Hi} n=85, EMT^{Lo} n=127 samples) following the ESTIMATE-based tumour purity algorithm. EMT^{Hi} and mesenchymal tumours were enriched for stromal cells. **** two-sided *p*-value < 0.0001. The comparison between the two groups was performed using an unpaired two-sided t test (**** *p*-value<0.0001). Data are shown as mean ±SEM. **C.** Kaplan-Meier survival analyses of the HNC patient groups were stratified based on their EMT ssGSEA score. The progression-free survival and disease-specific survival were significantly lower in EMT^{Hi} (n=85) compared to EMT^{Lo} (n=127) HNC patients while the stratification into disease-free survival based on the EMT ssGSEA score was not statistically significant. The comparison between the two groups was performed using a log-rank test (** *p*-value=0.0065, *** *p*-value=0.0003). **D.** Spearman's correlation matrix of the basal and mesenchymal TCGA-HNC subtypes (n=72). Source data are provided as a Source

19

Figure S2: Single cell analysis of patient HNC uncovered an epithelial-specific inverse correlation between partial EMT and PI3K signalling

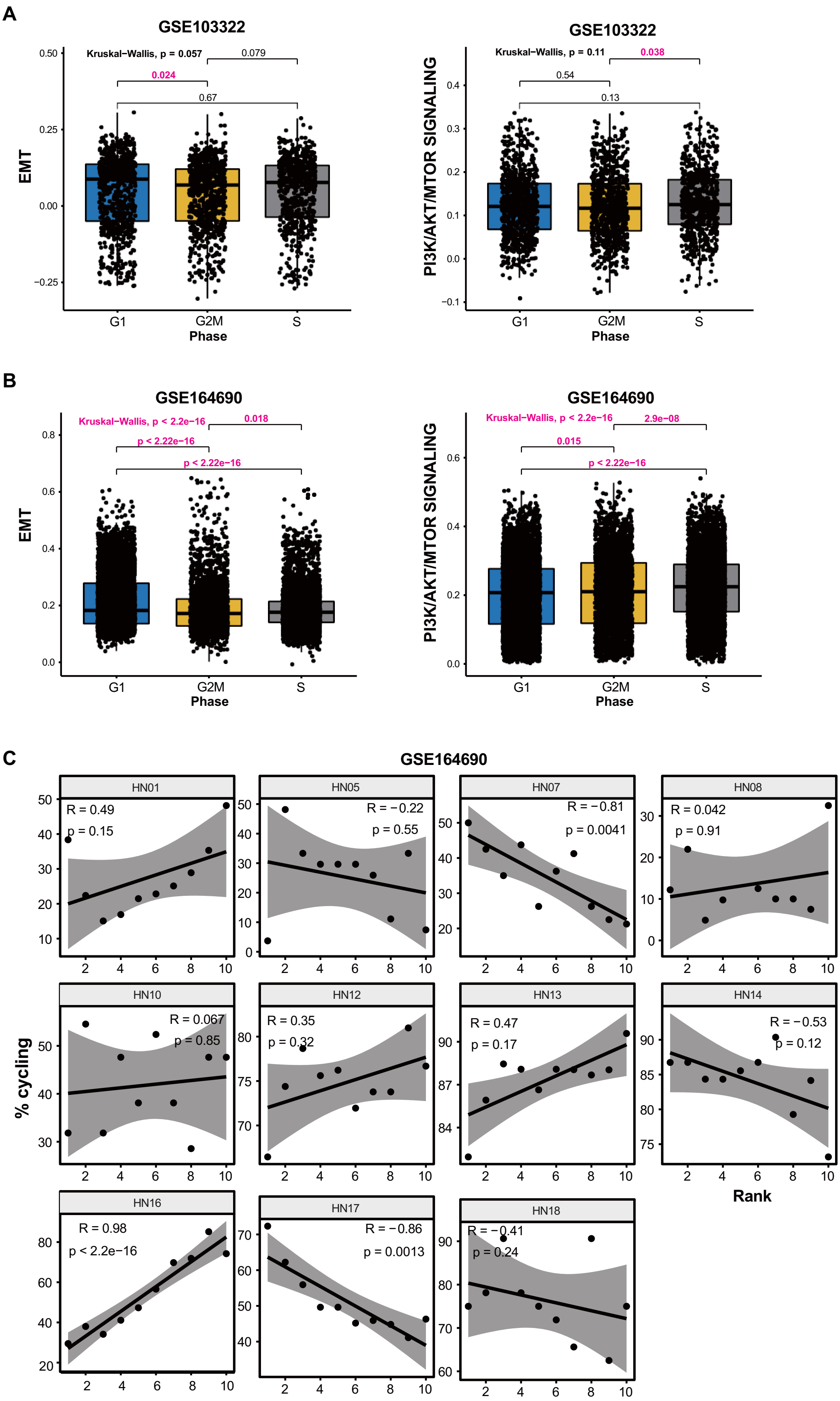

Figure S2. Single cell analysis of patient HNC uncovered an epithelial-specific inverse correlation between partial EMT and PI3K signalling. 2215 tumour cells from 10 HNC patients (GSE103322) and 13875 tumour cells from 11 HNC patients (GSE164690) were analyzed. **A-B.** Box plots for ssGSEA EMT scores are significantly higher in G1 compared to G2M/S phase indicating EMT is high in non-proliferating cells. Conversely, PI3K/AKT/mTOR hallmark gene set scores are significantly higher in S phase indicating cell proliferation. Comparisons between groups (GSE103322: G1=806, G2M=774, S=635 cells; GSE164690: G1=5906, G2M=2730, S=5239 cells) were performed using Kruskal-Wallis t test and unpaired two samples Wilcoxon test. Median values are shown in each box plot. All box plots include the median line, the box denotes the interquartile range (IQR), whiskers denote the rest of the data distribution and outliers are denoted by points greater than $\pm 1.5 \times$ IQR. **C.** Scatter graphs showing a decreasing percentage of cycling malignant cells and increasing p-EMT ssGSEA scores. The malignant cells are divided into ten sliding windows for each tumour. The consistent negative correlation was calculated using Spearman's correlation test. 95% confidence bands of the best-fit line are shown in gray. Source data are provided as a Source Data file.

Figure S3: Analysis of mutations in components of the PI3K pathway in the basal and mesenchymal subtypes

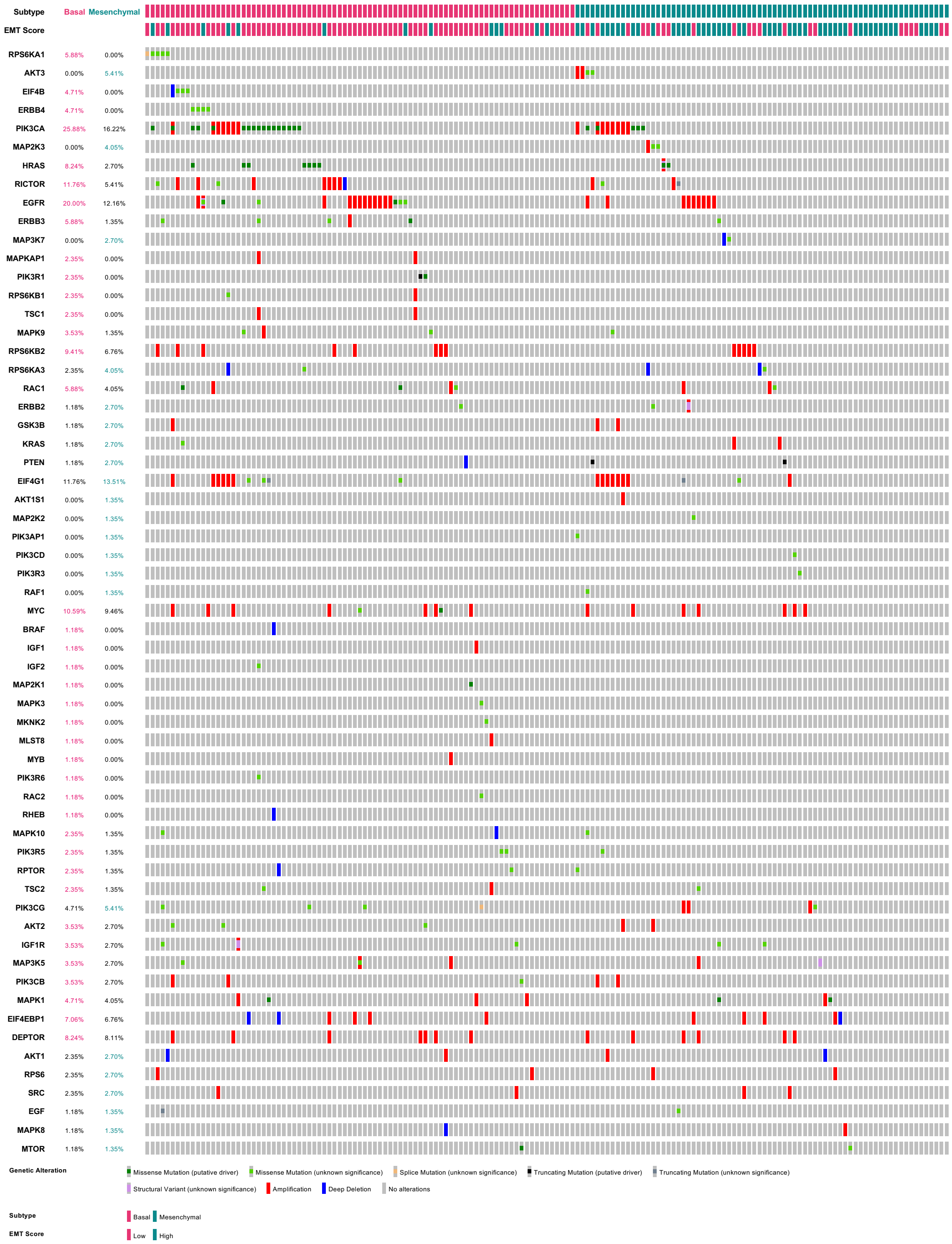

Figure S3: Analysis of mutations in components of the PI3K pathway in the basal and mesenchymal subtypes. OncoPrint of mutations in genes encoding components of the PI3K pathway in HNC tumours from basal (n=85) and mesenchymal (n=74) subtypes. Tumour subtypes and EMT ssGSEA scores are shown in the top panel. The frequency of mutations for each gene is listed on the left and the colours indicate a mutation-enriched group. Source data are provided as a Source Data file.

Figure S4: Pik3ca^{H1047R} knock-in onto a Grhl3^{cKO} background promotes basal-like HNC development in mice

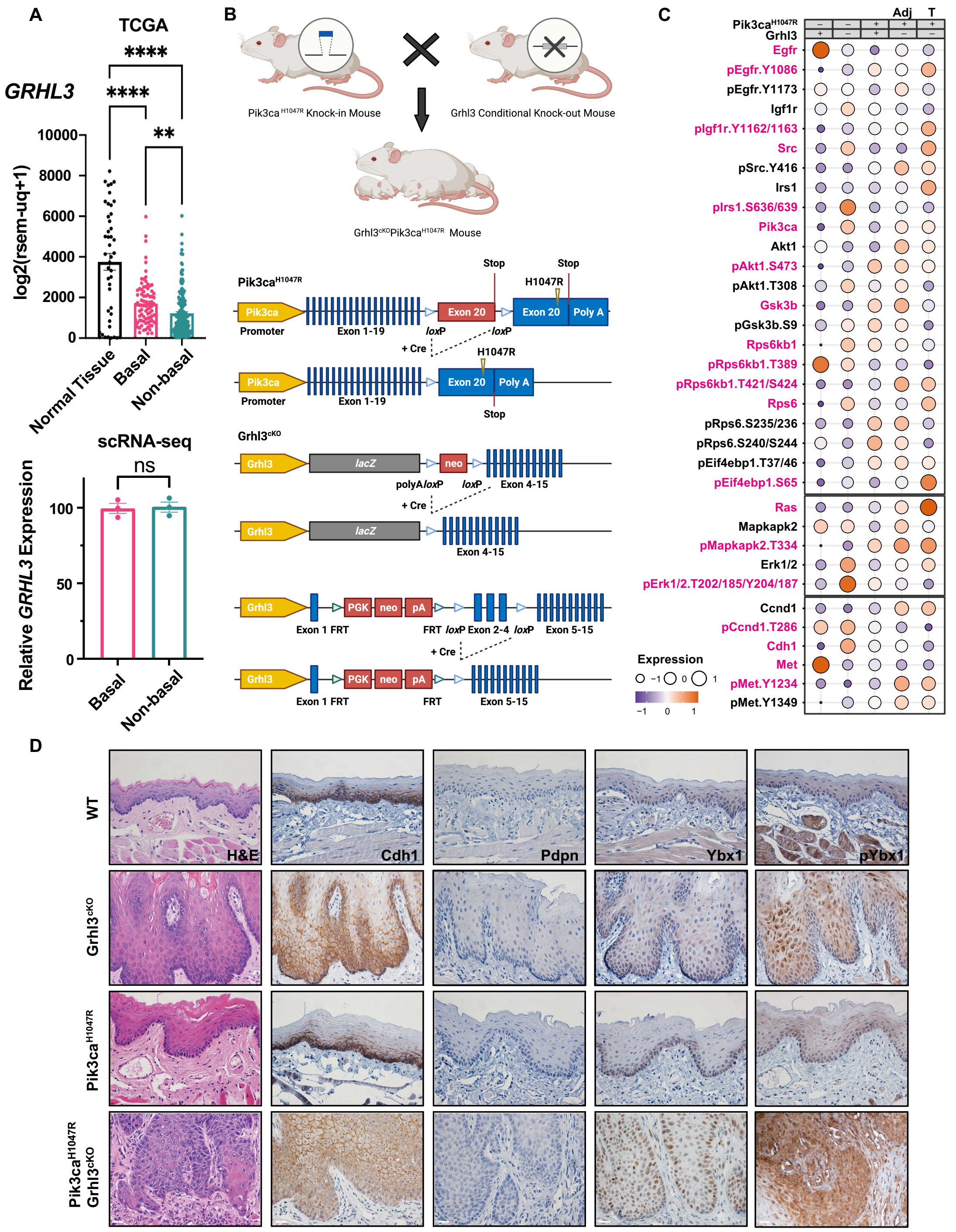

Figure S4. ***Pik3ca^{H1047R}*** **knock-in onto a** ***Grhl3*****-deficient background promotes basal-like HNC development in mice. A.** Analysis of *GRHL3* expression in primary basal (n=85) and non-basal (n=187) HNC compared to adjacent normal tissues (n=44) from the TCGA cohort (upper panel). The comparison between the groups was performed using an unpaired Student's t test (two-sided ** p=0.0098 and **** p<0.0001). Data are shown as mean ±SEM. GRHL3 levels were averaged from the 3 datasets of scRNA-seq (GSE103322, GSE164690 and GSE140042), showing a comparable level (ns, not significant) in clusters of epithelial cells of the basal subtype compared to non-basal

subtypes (lower panel). **B.** Schematic overview of the *Pik3ca*^{H1047R}*Grhl3*^{cKO} (K14-Cre) double mutant genetic mouse model of HNC. **C.** RPPA analyses of the PI3K/mTOR signalling and EMT markers in HNC cell lines. Expression levels are shown as relative fluorescence intensity values for differentially expressed proteins (one-way ANOVA p-value < 0.05) and highlighted in pink. Adjacent (Adj) tissue and tumour (T) samples are from the double mutant mice. Circles and colours represent the level of expression for each protein. **D.** H&E and IHC staining of CDH1, PDPN, YBX1 and phospho-YBX1 in single-mutant and *Pik3ca*^{H1047R}*Grhl3*^{cKO} mice. The epithelial CDH1 marker was expressed in tumours while the p-EMT marker PDPN was absent. YBX1 and phospho-YBX1 were expressed in basal proliferative cells. Magnification, X40; scale bars, 50µm. Supplementary Figure 4B was created using Biorender.com. Source data are provided as a Source Data file.

Figure S5: YBX1 phosphorylation is induced by activation of PI3K/mTOR signalling

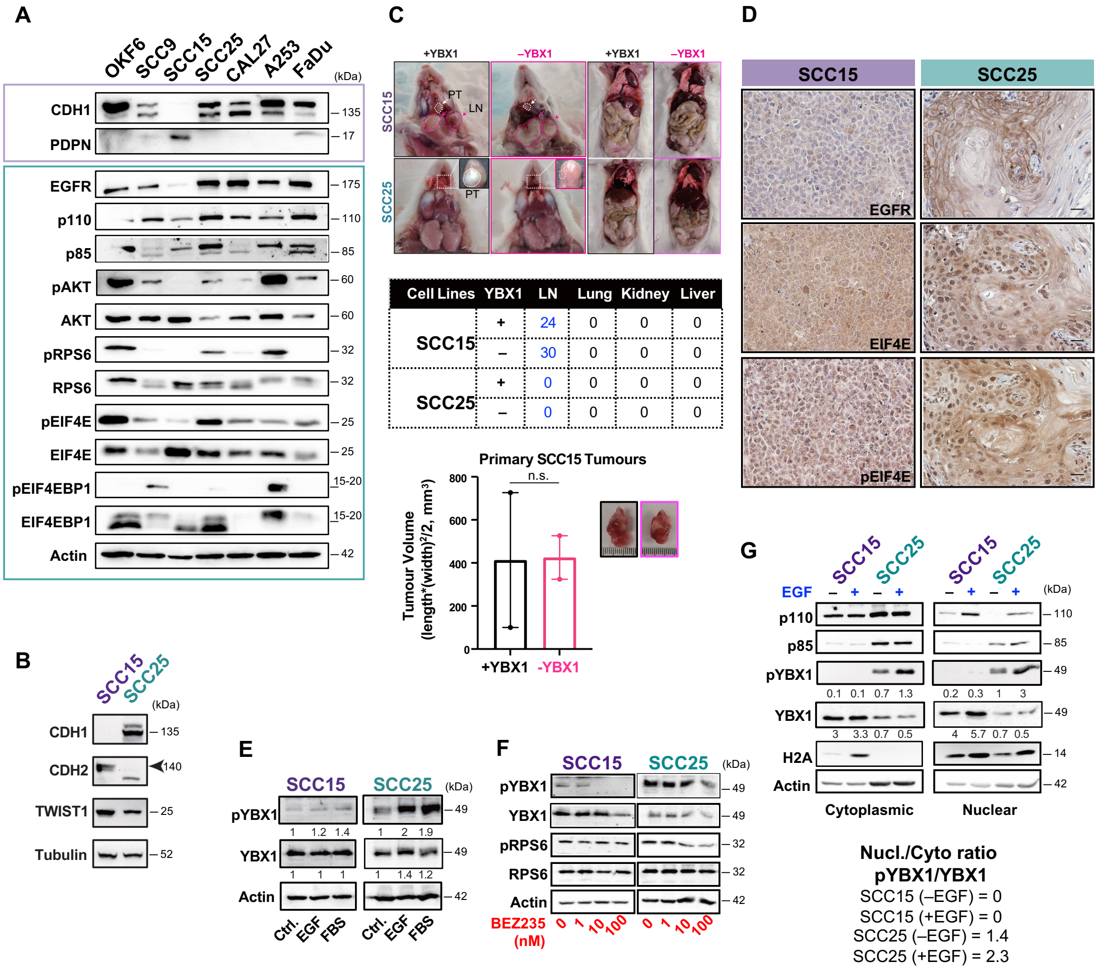

Figure S5. YBX1 phosphorylation is induced by activation of PI3K/mTOR signalling. A. WB analyses of PI3K/mTOR signalling and EMT markers in HNC cell lines. SCC15 cells showed inhibition of the PI3K signalling, PDPN expression but not CDH1, while SCC25 cells with active PI3K signalling expressed CDH1 but not PDPN. β-actin was used as the loading control. **B.** Additional EMT markers (N-cadherin and Twist1) show opposite expression pattern in SCC15 and SCC25. **C.** Representative images and summary of regional lymph node metastasis detected for SCC15 cells only even though SCC25 cells grew bigger primary tumours (upper panel). Lymph node metastasis for SCC15 +YBX1 (average 1.6 nodes/mouse) and for SCC15 –YBX1 (average 2 nodes/mouse). Distant metastasis in SCC15-grafted animals were not detected within the period of analysis (summary table). There was no significant difference in primary SCC15 +YBX1 and SCC15 –YBX1 tumour volume (lower panel) indicating that the tumour size did not correlate with regional lymph node metastasis (n=2 independent experiments). Data are shown as mean ±SEM. The comparison between the two groups was performed using an unpaired t test (n.s. not significant). **D.** IHC analysis of PI3K signaling components in orthotopic xenografts of SCC15 and SCC25. EGFR, EIF4E and pEIF4E were highly expressed in SCC25 tumours compared to SCC15. Magnification, X40; scale bars, 50μm. **E.** 100 ng/mL EGF or 20% FBS for 30 min treatments induced

phosphorylation of YBX1 in SCC25 and a slight increase in p-YBX1 in SCC15 as shown by protein quantification. **F.** The inhibition of PI3K/mTOR signalling reduced p-YBX1 in both SCC15 and SCC25 treated with 100nM of BEZ235 for 6 hours. **G.** WB analysis of p-YBX1 and YBX1 in cytoplasmic and nuclear fractions of cells treated with EGF (100 ng/mL). p-YBX1 was detected in both the cytoplasmic and nuclear fractions of invasive SCC15 at low levels, and at high levels in proliferative SCC25. The nuclear to cytoplasmic ratio of p-YBX1/YBX1 indicates that EGF treatment promoted nuclear localisation of p-YBX1 in SCC25 but not in SCC15 cells. WB in **A, B, E–G** are representative of n=2 biological replicates. Source data are provided as a Source Data file.

Figure S6: Loss of YBX1 in mesenchymal HNC induces resistance to BEZ235

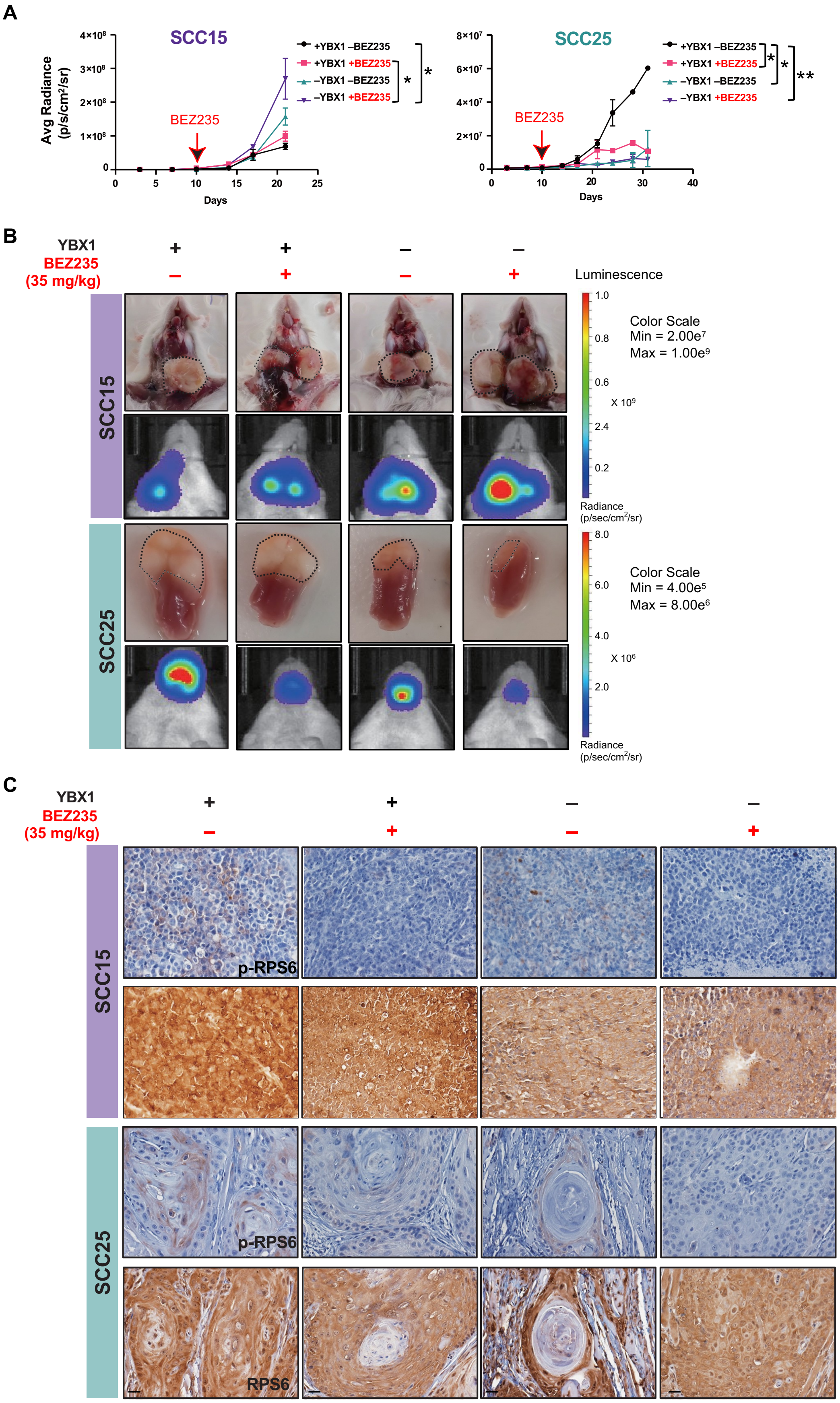

96 **Figure S6: Loss of YBX1 in mesenchymal HNC induces resistance to BEZ235. A.**

Bioluminescence IVIS imaging of live NSG animals implanted with either SCC25 or SCC15 (n=2 experiments, 3 mice/group/experiment). SCC25 –YBX1 showed reduced tumour growth with no significant response to BEZ235 treatment (35mg/kg, daily gavage for 4 weeks) compared to SCC25 +YBX1. SCC15 –YBX1 showed a growth advantage over SCC15 +YBX1 and increased resistance to BEZ235 treatment. The radiance of bioluminescence was measured weekly and at sacrifice. Red arrows indicate the starting day of BEZ235 administration. Data are shown as mean ±SEM at each timepoint. **B.** Representative images showing optical macroscopy of the mice at necropsy and their luminescence images. Statistical significance was assessed using the unpaired two-sided Student's t test. For SCC15, statistical significance for comparisons between –YBX1 +BEZ235 and +YBX1 +BEZ235 was p=0.024, and +YBX1 –BEZ235 was p=0.014. For SCC25, statistical significance for comparisons between +YBX1 –BEZ235 and +YBX1 +BEZ235 was p=0.02, and –YBX1 –BEZ235 was p=0.013, and –YBX1 +BEZ235 was p=0.009. **C.** IHC for p-RPS6 and total RPS6 demonstrating BEZ235 efficacy in SCC15 and SCC25 xenografts. Magnification, X40; scale bars, 50µm. Source data are provided as a Source Data file.

Figure S7: YBX1 nuclear localisation reverses cell invasion

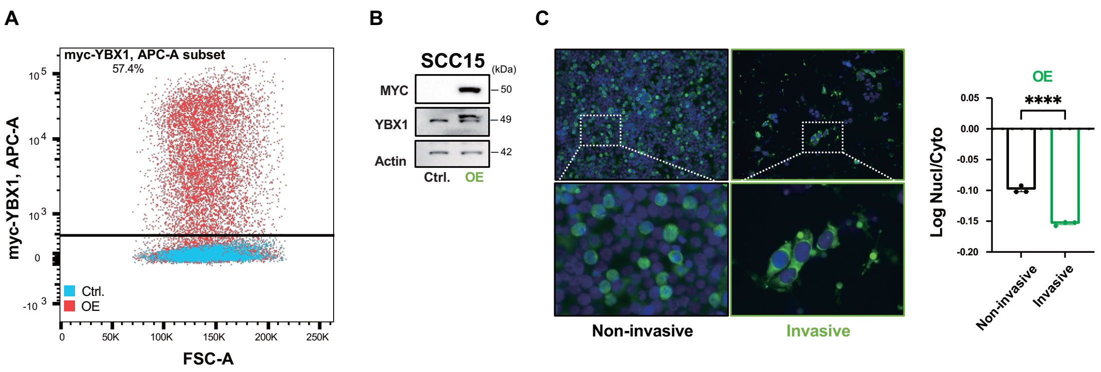

Figure S7: YBX1 nuclear localisation reverses cell invasion: A. The overexpression (OE) of myc-tagged YBX1 in SCC15 is shown using flow cytometry (54.7% transfection rate) and (**B**) by anti-MYC and anti-YBX1 western blots. **C.** Representative images of the transwell invasion assay showing YBX1 OE cells with nuclear YBX1 expression in green (IF for the MYC tag) are retained in the inner membrane (non-invasive) whether SCC15 cells with cytoplasmic YBX1 are detected in the outer membrane (invasive). The quantification of YBX1 OE using CellProfiler demonstrated reduction of the nuclear to cytoplasmic ratio and indicates that OE of YBX1 can localise to the nucleus of SCC15 to inhibit cell invasion. Magnification, X40; scale bars, 50µm. Data are shown as mean ±SEM. The comparison between the two groups was performed using an unpaired t test (**** p-value<0.0001, n=3 experiments). WB in **B** are representative of n=2 biological replicates. FACS gating strategies and source data are provided as a Source Data file.

Figure S8: Expression of pYBX1 and PDPN in patient HNC samples

A

		Patient No. (n=135)
**Differentiation Grading**		
	Well	51
	Moderate	60
	Poor	23
**Ki67**		
	0	13
	1	67
	2	37
	3	6
	4	12
**p16**		
	Negative	111
	Positive	24
**p53**		
	Negative	68
	Positive	48
	Overexpression	19

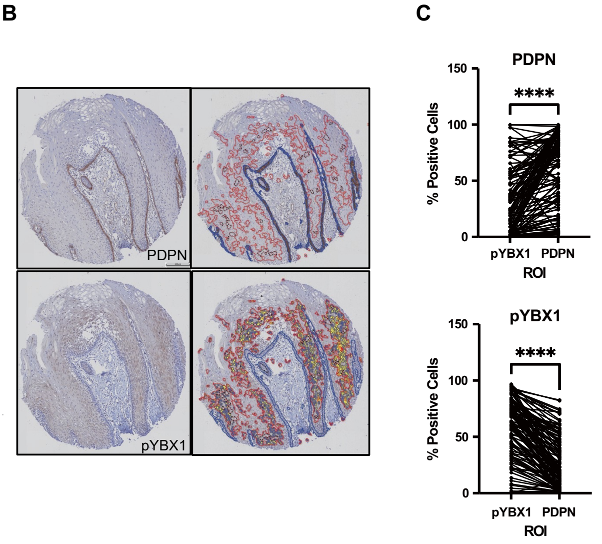

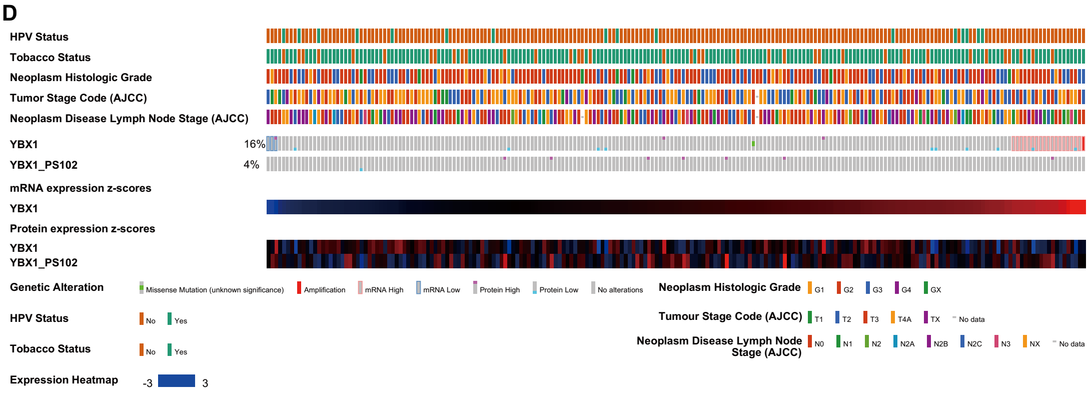

Figure S8: Expression of pYBX1 and PDPN in patient HNC samples. A. Pathological and molecular characteristics of the patient samples (n=135) from the HNC tissue microarray (TMA). **B.** Representative images of pYBX1 and PDPN region of interests (ROIs) in serial sections used for the quantification of IHC staining. HALO quantitative image analysis classified the pYBX1 and PDPN positive ROI staining areas using random forest. pYBX1 and PDPN positive cells were counted within the ROIs and shown in red and brown, respectively. **C.** The percentage of pYBX1/PDPN positive cells in the ROIs indicates a significantly inversed correlation across the n=135 patient samples. The comparison between the two groups was performed using a paired t test (**** p<0.0001). **D.** The clinicopathological annotations of HNC primary tumours (upper, n=211) and YBX1 mutations, mRNA and protein expression (lower) extracted from OncoPrint depict the YBX1 genetic alterations in human HNC.

Supplementary Table_Antibodies list					
**Antibody**	**Company**	**Catalogue No.**	**Clone**	**Dilution**	**Assay**
Actin	Abcam	ab8229		1:3000	WB
CDH1	Cell Signaling Technology	CST3195	24E10	1:1000, 1:200	WB, IHC
PDPN	Abcam	ab128994	EPR7072	1:1000, 1:200	WB, IHC
EGFR	Cell Signaling Technology	CST4267	D38B1	1:1000, 1:50	WB, IHC
p110	Cell Signaling Technology	CST4249	C73F8	1:1000	WB
p85	Cell Signaling Technology	CST4257	19H8	1:1000	WB
pAKT (Ser473)	Cell Signaling Technology	CST9271		1:1000	WB
AKT	Cell Signaling Technology	CST9272		1:1000	WB
pRPS6 (Ser240/244)	Cell Signaling Technology	CST2215		1:1000	WB
RPS6	Cell Signaling Technology	CST2217	5G10	1:1000	WB
pEIF4E (Ser209)	Abcam	ab76256	EP2151Y	1:1000, 1:100	WB, IHC
EIF4E	Cell Signaling Technology	CST9742		1:1000, 1:50	WB, IHC
pEIF4EBP1	Cell Signaling Technology	CST9451		1:1000	WB
EIF4EBP1	Cell Signaling Technology	CST9644	53H11	1:1000	WB
CDH2	Thermo Fisher Scientific	PA5-17526		1:1000	WB
TWIST1	Thermo Fisher Scientific	711565		1:1000	WB
Tubulin	Cell Signaling Technology	CST3873	DM1A	1:3000	WB
pYBX1 (Ser102)	Cell Signaling Technology	CST2900	C34A2	1:1000, 1:200, 1:200	WB, IHC, IF
YBX1	Cell Signaling Technology	CST4202		1:1000, 1:50, 1:50	WB, IHC, IF
H2A	Cell Signaling Technology	CST12349	D6O3A	1:1000	WB
GFP	Santa Cruz Biotechnology	sc-9996	B-2	1:50	IF
MYC tag	Cell Signaling Technology	CST47029	9B11	1:1000; 1:50	WB, FACS
Goat-anti-rabbit 647	Abcam	ab150079		1:500	IF
Goat-anti-rabbit 488	Abcam	ab150077		1:500	IF
Goat-anti-mouse 488	Abcam	ab150113		1:200	IF
Goat-anti-rabbit-HRP	Bio-rad	1706515		1:1000	WB
Goat-anti-mouse-HRP	Bio-rad	1706516		1:1000	WB
