# Supplementary material for: YBX1 integration of oncogenic PI3K/mTOR signalling regulates the fitness of malignant epithelial cells
Source: Nat Commun. 2023 Mar 22;14:1591. doi: 10.1038/s41467-023-37161-0 (PMC10033729; doi:10.1038/s41467-023-37161-0)
Supplement: Supplementary file 3 — Reporting Summary [file 41467_2023_37161_MOESM3_ESM.pdf]

nature portfolio

Corresponding author(s): Charbel Darido

Last updated by author(s): Mar 1, 2023

Reporting Summary

Nature Portfolio wishes to improve the reproducibility of the work that we publish. This form provides structure for consistency and transparency in reporting. For further information on Nature Portfolio policies, see our Editorial Policies and the Editorial Policy Checklist.

Statistics

For all statistical analyses, confirm that the following items are present in the figure legend, table legend, main text, or Methods section.

n/a	Confirmed	
☐	☒	The exact sample size (*n*) for each experimental group/condition, given as a discrete number and unit of measurement
☐	☒	A statement on whether measurements were taken from distinct samples or whether the same sample was measured repeatedly
☐	☒	The statistical test(s) used AND whether they are one- or two-sided<br>*Only common tests should be described solely by name; describe more complex techniques in the Methods section.*
☐	☒	A description of all covariates tested
☐	☒	A description of any assumptions or corrections, such as tests of normality and adjustment for multiple comparisons
☐	☒	A full description of the statistical parameters including central tendency (e.g. means) or other basic estimates (e.g. regression coefficient) AND variation (e.g. standard deviation) or associated estimates of uncertainty (e.g. confidence intervals)
☐	☒	For null hypothesis testing, the test statistic (e.g. *F*, *t*, *r*) with confidence intervals, effect sizes, degrees of freedom and *P* value noted<br>*Give P values as exact values whenever suitable.*
☒	☐	For Bayesian analysis, information on the choice of priors and Markov chain Monte Carlo settings
☒	☐	For hierarchical and complex designs, identification of the appropriate level for tests and full reporting of outcomes
☐	☒	Estimates of effect sizes (e.g. Cohen's *d*, Pearson's *r*), indicating how they were calculated

Our web collection on statistics for biologists contains articles on many of the points above.

Software and code

Policy information about availability of computer code

Data collection	No software was used for data collection.
Data analysis	Statistical significance was assessed using the unpaired Student's t test, one-way ANOVA, Spearman's correlation test, two-sided Wald test for multivariate Cox regression analysis or log-rank test for survival analysis using Prism 9 (GraphPad). Statistical analyses for the RPPA, RNA sequencing and single-cell RNA sequencing were carried out using R version 4.0.5. Packages used for single-cell RNA sequencing analysis included Cell Ranger pipeline v.3.0.2, Seurat_4.1.0; SummarizedExperiment_1.20.0; clustree_0.4.4; escape_1.3.3; SingleR_1.8.1. Packages used for RNA sequencing analysis included limma_3.46.0, GSEA_v4.3.2 and Galaxy suie environemtn (version 4.0, including Cutapdapt, HISAT2, featureCounts and Limma). Packages used for plotting included tidyverse_1.3.1, ComplexHeatmap_2.8.0 and ggpubr_0.4.0. The flow cytometry data was analyzed using FACSDiva 9.0. SPOT software 5.0, CellProfiler_4.2.4 and HALO quantitative image analysis platform 2.0 were used for IHC image analysis.

For manuscripts utilizing custom algorithms or software that are central to the research but not yet described in published literature, software must be made available to editors and reviewers. We strongly encourage code deposition in a community repository (e.g. GitHub). See the Nature Portfolio guidelines for submitting code & software for further information.

Data

Policy information about availability of data

All manuscripts must include a data availability statement. This statement should provide the following information, where applicable:

- Accession codes, unique identifiers, or web links for publicly available datasets
- A description of any restrictions on data availability
- For clinical datasets or third party data, please ensure that the statement adheres to our policy

The data that support this study are available in this paper and stored in GEO database as SuperSeries GSE226357 (“GSE226357[https://www.ncbi.nlm.nih.gov/geo/query/acc.cgi?acc=GSE226357]”, including RNAseq raw data (“GSE226355[https://www.ncbi.nlm.nih.gov/geo/query/acc.cgi?acc=GSE226355]”), and RPPA raw data (“GSE226356[https://www.ncbi.nlm.nih.gov/geo/query/acc.cgi?acc=GSE226356]”) or from the corresponding author (charbel.darido@petermac.org) upon reasonable request. Publicly available data from TCGA, Broad Institute and Stanford University were used. TCGA genetic, transcriptomic, proteomic, and clinical data were downloaded from the cBioportal data portal (https://www.cbioportal.org/; Head and Neck Squamous Cell Carcinoma (TCGA, PanCancer Atlas)). Genetic and transcriptomic data from Broad Institute were downloaded from depmap portal (https://depmap.org/portal/; CCLE_expression.csv; CCLE_mutation.csv). The single-cell RNA sequencing data (GSE140042, GSE103322, GSE164690) were downloaded from GEO database (https://www.ncbi.nlm.nih.gov/gds). Raw data for bulk mRNA-seq on mouse tissues and human cancer cell lines and RPPA data from different conditions have been deposited on the Figshare repository. Raw data on mouse tissues and human cancer cell lines from different conditions are available on figshare; for bulk mRNA-seq (“20024246[https://doi.org/10.6084/m9.figshare.20024246.v2]”) and for RPPA (“20024258[https://doi.org/10.6084/m9.figshare.20024258.v2]”). Public single-cell RNA-seq data on patient samples were downloaded from GSE140042, GSE103322 and GSE164690. The processed single-cell RNA-seq data is available on figshare (“20033024[https://doi.org/10.6084/m9.figshare.20033024.v2]”).
Additional data are available as supplementary materials and source data as Source Data files.

Human research participants

Policy information about studies involving human research participants and Sex and Gender in Research.

Reporting on sex and gender	N/A
Population characteristics	N/A
Recruitment	No patients were recruited as part of this study.
Ethics oversight	Ethical approval number RA/4/1/8562

Note that full information on the approval of the study protocol must also be provided in the manuscript.

Field-specific reporting

Please select the one below that is the best fit for your research. If you are not sure, read the appropriate sections before making your selection.

☒ Life sciences ☐ Behavioural & social sciences ☐ Ecological, evolutionary & environmental sciences

For a reference copy of the document with all sections, see nature.com/documents/nr-reporting-summary-flat.pdf

Life sciences study design

All studies must disclose on these points even when the disclosure is negative.

Sample size	The analysis was performed on available head and neck cancer samples with genetic, transcriptomic and proteomic profiles from TCGA and all tumour samples that passed quality control from GSE140042, GSE103322, GSE16490 for single-cell RNA sequencing. A minimum sample size of 3 replicates was considered as sufficient for RNA-seq and 2 replicates for RPPA analyses in cell lines and 3 replicates for RPPA analysis of mouse tissues. The replicate number was satisfactory to achieve a power between 0.8 and 1 as described in Ching, et. al, Power analysis and sample size estimation for RNA-seq differential expression. RNA, 2014.
Data exclusions	For single-cell RNA sequencing, only samples with 2,000 to 4,000 sequenced cells were considered for downstream analyses while others were excluded. Cells with more than 200 RNA features were retained and others removed. RNA features detected in more than 2 cells were kept.
Replication	Independent in vitro experiments (e.g. colony formation and invasion assays, immunofluorescence) were repeated a minimum of three times, analyzed and confirmed by independent researchers. In vivo experiments (e.g. growth of xenografts) were independently repeated twice for each cell line. RNA-sequencing (3 replicates per cell line); RPPA (2 replicates per human cell line and 3 replicates per mouse tissue). The replicates are all reproducible.

Randomization	No randomization was used in this study due to the small sample size. Covariates were controlled by running controls in parallel whenever applicable. Appropriate controls were used throughout the study.
Blinding	Blinding was not relevant for the bioinformatics analyses because our aims were based on objective computation results rather than human labels. Blinding for in vitro experiments was impractical. Blinding the in vivo experiments was not feasible given the study design, the ethical monitoring and labeling requirements of the animal facility.

Reporting for specific materials, systems and methods

We require information from authors about some types of materials, experimental systems and methods used in many studies. Here, indicate whether each material, system or method listed is relevant to your study. If you are not sure if a list item applies to your research, read the appropriate section before selecting a response.

Materials & experimental systems

n/a	Involved in the study
☐	☒ Antibodies
☐	☒ Eukaryotic cell lines
☒	☐ Palaeontology and archaeology
☐	☒ Animals and other organisms
☒	☐ Clinical data
☒	☐ Dual use research of concern

Methods

n/a	Involved in the study
☒	☐ ChIP-seq
☐	☒ Flow cytometry
☒	☐ MRI-based neuroimaging

Antibodies

Antibodies used

Actin Abcam ab8229, CDH1 Cell Signaling Technology CST3195, PDPN Abcam ab128994, EGFR Cell Signaling Technology CST4267, p110 Cell Signaling Technology CST4249, p85 Cell Signaling Technology CST4257, pAKT (Ser473) Cell Signaling Technology CST9271, AKT Cell Signaling Technology CST9272, pRPS6 (Ser240/244) Cell Signaling Technology CST2215, RPS6 Cell Signaling Technology CST2217, pEIF4E (Ser209) Abcam ab76256, EIF4E Cell Signaling Technology CST9742, pEIF4EBP1 Cell Signaling Technology CST9451, EIF4EBP1 Cell Signaling Technology CST9644, CDH2 Thermo Fisher Scientific PA5-17526, TWIST1 Thermo Fisher Scientific 711565, Tubulin Cell Signaling Technology CST3873, pYBX1 (Ser102) Cell Signaling Technology CST2900, YBX1 Cell Signaling Technology CST4202, H2A Cell Signaling Technology CST12349, MYC tag Cell Signaling Technology CST47029, Goat-anti-rabbit-HRP Bio-rad 1706515, Goat-anti-mouse-HRP Bio-rad 1706516 were used for western blot and IHC analyses. pYBX1 (Ser102) Cell Signaling Technology CST2900, YBX1 Cell Signaling Technology CST4202, GFP Santa Cruz Biotechnology sc-9996, Goat-anti-rabbit 647 Abcam ab150079, Goat-anti-rabbit 488 Abcam ab150077, Goat-anti-mouse 488 Abcam ab150113 were used for IF. MYC tag-APC CST#47029 was used for flow cytometry and western blot. Antibodies used for RPPA analysis are listed in the Source Data file.

Validation

All antibodies are commercial and have been validated by their respective manufacturers.
Antibody Company Catalogue No. Clone Dilution Assay URL
Actin Abcam ab8229 1:3000 WB https://www.abcam.com/beta-actin-antibody-loading-control-ab8229.html
CDH1 Cell Signaling Technology CST3195 24E10 1:1000, 1:200 WB, IHC https://www.cellsignal.com/products/primary-antibodies/e-cadherin-24e10-rabbit-mab/3195
PDPN Abcam ab128994 EPR7072 1:1000, 1:200 WB, IHC https://www.abcam.com/podoplanin--gp36-antibody-epr7072-ab128994.html
EGFR Cell Signaling Technology CST4267 D38B1 1:1000, 1:50 WB, IHC https://www.cellsignal.com/products/primary-antibodies/egf-receptor-d38b1-xp-rabbit-mab/4267
p110 Cell Signaling Technology CST4249 C73F8 1:1000 WB https://www.cellsignal.com/products/primary-antibodies/pi3-kinase-p110a-c73f8-rabbit-mab/4249
p85 Cell Signaling Technology CST4257 19H8 1:1000 WB https://www.cellsignal.com/products/primary-antibodies/pi3-kinase-p85-19h8-rabbit-mab/4257
pAKT (Ser473) Cell Signaling Technology CST9271 1:1000 WB https://www.cellsignal.com/products/primary-antibodies/phospho-akt-ser473-antibody/9271
AKT Cell Signaling Technology CST9272 1:1000 WB https://www.cellsignal.com/products/primary-antibodies/akt-antibody/9272
pRPS6 (Ser240/244) Cell Signaling Technology CST2215 1:1000 WB https://www.cellsignal.com/products/primary-antibodies/phospho-s6-ribosomal-protein-ser240-244-antibody/2215
RPS6 Cell Signaling Technology CST2217 5G10 1:1000 WB https://www.cellsignal.com/products/primary-antibodies/s6-ribosomal-protein-5g10-rabbit-mab/2217
pEIF4E (Ser209) Abcam ab76256 EP2151Y 1:1000, 1:100 WB, IHC https://www.abcam.com/eif4e-phospho-s209-antibody-ep2151y-ab76256.html
EIF4E Cell Signaling Technology CST9742 1:1000, 1:50 WB, IHC https://www.cellsignal.com/products/primary-antibodies/eif4e-antibody/9742
pEIF4EBP1 Cell Signaling Technology CST9451 1:1000 WB https://www.cellsignal.com/products/primary-antibodies/phospho-4e-bp1-ser65-antibody/9451
EIF4EBP1 Cell Signaling Technology CST9644 53H11 1:1000 WB https://www.cellsignal.com/products/primary-antibodies/4e-bp1-53h11-rabbit-mab/9644
CDH2 Thermo Fisher Scientific PA5-17526 1:1000 WB https://www.thermofisher.com/antibody/product/N-cadherin-Antibody-Polyclonal/PA5-17526
TWIST1 Thermo Fisher Scientific 711565 1:1000 WB https://www.thermofisher.com/antibody/product/TWIST1-Antibody-Recombinant-Polyclonal/711565

Tubulin Cell Signaling Technology CST3873 DM1A 1:3000 WB https://www.cellsignal.com/products/primary-antibodies/a-tubulin-dm1a-mouse-mab/3873?site-search-type=Products&N=4294956287&Ntt=a-tubulin&fromPage=plp
pYBX1 (Ser102) Cell Signaling Technology CST2900 C34A2 1:1000, 1:200, 1:200 WB, IHC, IF https://www.cellsignal.com/products/primary-antibodies/phospho-yb1-ser102-c34a2-rabbit-mab/2900
YBX1 Cell Signaling Technology CST4202 1:1000, 1:50, 1:50 WB, IHC, IF https://www.cellsignal.com/products/primary-antibodies/yb1-d299-antibody/4202
H2A Cell Signaling Technology CST12349 D6O3A 1:1000 WB https://www.cellsignal.com/products/primary-antibodies/histone-h2a-d6o3a-rabbit-mab/12349?site-search-type=Products&N=4294956287&Ntt=h2a&fromPage=plp
GFP Santa Cruz Biotechnology sc-9996 B-2 1:50 IF https://www.scbt.com/p/gfp-antibody-b-2
MYC tag Cell Signaling Technology CST47029 9B11 1:1000; 1:50 WB, FACS https://www.cellsignal.com/products/antibody-conjugates/myc-tag-9b11-mouse-mab-apc-conjugate/47029
Goat-anti-rabbit 647 Abcam ab150079 1:500 IF https://www.abcam.com/goat-rabbit-igg-hl-alexa-fluor-647-ab150079.html
Goat-anti-rabbit 488 Abcam ab150077 1:500 IF https://www.abcam.com/goat-rabbit-igg-hl-alexa-fluor-488-ab150077.html
Goat-anti-mouse 488 Abcam ab150113 1:200 IF https://www.abcam.com/goat-mouse-igg-hl-alexa-fluor-488-ab150113.html
Goat-anti-rabbit-HRP Bio-rad 1706515 1:1000 WB https://www.bio-rad.com/en-au/sku/1706515-goat-anti-rabbit-igg-h-l-hrp-conjugate?ID=1706515
Goat-anti-mouse-HRP Bio-rad 1706516 1:1000 WB https://www.bio-rad.com/en-au/sku/1706516-goat-anti-mouse-igg-h-l-hrp-conjugate?ID=1706516

Eukaryotic cell lines

Policy information about cell lines and Sex and Gender in Research

Cell line source(s)	Lenti-X-293T (#632180) from Takara Bio<br>OKF6 from Harvard Skin Disease Research.<br>SCC9 (CRL-1629), SCC15 (CRL-1623), SCC25 (CRL-1628), CAL27 (CRL-2095), A253 (HTB-41) and FaDu (HTB-43) from the American Type Culture Collection (ATCC).
Authentication	All cell lines were authenticated by ATCC and validated by short tandem repeat profiling in house.
Mycoplasma contamination	All cell lines were tested negative fro mycoplasma contamination using PCR.
Commonly misidentified lines<br>(See ICLAC register)	Commonly misidentified cell lines were not used in this study.

Animals and other research organisms

Policy information about studies involving animals; ARRIVE guidelines recommended for reporting animal research, and Sex and Gender in Research

Laboratory animals	Pik3caH1047R-Grhl3cKO mice were generated by crossing Keratin 14-Cre mice with Pik3ca flox/+ /Grhl3 flox/– mice (C57BL/6J background) and males and females equally used at 3 months old as described in Figure S4B.<br>6-8 weeks NSG mice were purchased from Jackson Laboratory (#005557). Mice were housed under 12 hours light-dark cycles at 20-26C and 20-70% humidity, in ventilated cages and constant access to food and water.
Wild animals	This study did not involve wild animals.
Reporting on sex	Both female and male mice were used in this study. Sex-based analysis was not conducted.
Field-collected samples	This study did not involve samples collected from the field.
Ethics oversight	All animal studies including breeding, experiments and euthanasia protocols were performed in accordance with the National Code of Practice for the Care and Use of Animals for Scientific Purposes and the Animal Ethics guidelines, and were approved by the institutional Animal Experimentation Ethics Committee (E587 and E632) at the Peter MacCallum Cancer Center.

Note that full information on the approval of the study protocol must also be provided in the manuscript.

Flow Cytometry

Plots

Confirm that:

☒ The axis labels state the marker and fluorochrome used (e.g. CD4-FITC).

☒ The axis scales are clearly visible. Include numbers along axes only for bottom left plot of group (a 'group' is an analysis of identical markers).

☒ All plots are contour plots with outliers or pseudocolor plots.

☒ A numerical value for number of cells or percentage (with statistics) is provided.

Methodology

Sample preparation	Cells were collected and stained with 1:200 anti-MYC-APC antibody according to BD Cytofix/Cytoperm kit manual. Data were collected using the BD FACSCanto II (FACSDiva 9.0)
Instrument	BD FACSCanto II
Software	FACSDiva 9.0
Cell population abundance	Cell population were analyses based on fluorescence intensity and not population site.
Gating strategy	Cells were intitially gates based on FSC-A vs SSC-A, followed by FSC-A vs FSC-H to obtain single cells. Dead cells were excluded based on DAPI fluoresence. Where appropriate, cells with higher APC intensity compared to control cells were considered as the positive ones.

☒ Tick this box to confirm that a figure exemplifying the gating strategy is provided in the Supplementary Information.
